# Supplementary material for: Entropy production bounds for systems running computer programs
Source: PNAS Nexus. 2026 Apr 16;5(4):pgag116. doi: 10.1093/pnasnexus/pgag116 (PMC13101992; doi:10.1093/pnasnexus/pgag116)
Supplement: pgag116_Supplementary_Data [file pgag116_supplementary_data.pdf]

Supplementary information for "Entropy production bounds for systems running computer programs"

Abhishek Yadav[ab*], Francesco Caravelli[c], and David Wolpert[adef]

[a]Santa Fe Institute, 1399 Hyde Park Road Santa Fe, NM 87501, USA
[b]Department of Physical Sciences, IISER Kolkata, Mohanpur 741246, India
[c]Theoretical Division (T-4), Los Alamos National Laboratory, New Mexico, 87545, USA
[d]Complexity Science Hub, Vienna, Austria
[e]Arizona State University, Tempe, AZ 85281, USA
[f]International Center for Theoretical Physics, Trieste 34151, Italy and
[*]Corresponding author: abhi.yadav.28k@gmail.com

I. METHODS OF ESTIMATION OF PRIOR DISTRIBUTION

Consider a system with discrete space $\mathcal{X}$ and let $\Delta_{\mathcal{X}}$ denote the simplex on the state space. Suppose that an initial distribution $p_{X_0} \in \Delta_{\mathcal{X}}$ transforms to a final distribution $p_{X_1} \in \Delta_{\mathcal{X}}$, where X_0 and X_1 are random variables representing system's state at initial and final time respectively, such that the transformation can be expressed by a conditional distribution $G(x|x')$ specifying the probability of a final state given an inital state:

$$p_{X_1}(x) = \sum_{x' \in \mathcal{X}} G(x|x') p_{X_0}(x') \tag{1}$$

or $p_{X_1} = G p_{X_0}$ as short hand. For a real valued function $f : \mathcal{X} \to \mathbb{R}$, consider a cost function of the form,

$$\mathcal{C}(p_{X_0}) = S\left(G p_{X_0}\right) - S\left(p_{X_0}\right) + \langle f \rangle_{p_{X_0}}. \tag{2}$$

Define an equivalence relation on the state space $\mathcal{X}$ as follows:

$$x \sim x' \iff \exists y \in \mathcal{X} \text{ such that } G(y|x) > 0 \text{ and } G(y|x') > 0. \tag{3}$$

Let $\mathcal{L}_G(\mathcal{X})$ denote the partition of $\mathcal{X}$ induced by the transitive closure of this equivalence relation. This partition is referred to as the island decomposition of $\mathcal{X}$ under the map G, and each subset $\mathcal{Z} \in \mathcal{L}_G(\mathcal{X})$ is called an island of $\mathcal{X}$ with respect to G.

It is known that if G induces a single island—i.e., $\mathcal{L}_G(\mathcal{X}) = \{\mathcal{X}\}$—then the minimizer of the cost function $\mathcal{C}$ in Eq. 2, given by $q_{X_0} = \arg\min_{r_0} \mathcal{C}(r_0)$, is unique [1].

However, when the map G induces multiple islands—each indexed by c and denoted by $\mathcal{Z}_c$—we can define, for any distribution $p_{X_0} \in \Delta_{\mathcal{X}}$, the conditional distribution over each island as follows:

$$p_{X_0|c}(x) = \begin{cases} \dfrac{p_{X_0}(x)}{p(c)} & \text{if } x \in \mathcal{Z}_c, \\ 0 & \text{otherwise.} \end{cases} \tag{4}$$

where $p(c) = \sum_{x \in \mathcal{Z}_c} p_{X_0}(x)$ is the total probability mass assigned to island $\mathcal{Z}_c$. The corresponding cost function restricted to island $\mathcal{Z}_c$ is then given by:

$$\mathcal{C}(p_{X_0|c}) = S\left(G p_{X_0|c}\right) - S\left(p_{X_0|c}\right) + \langle f \rangle_{p_{X_0|c}}. \tag{5}$$

Furthermore, the total cost can be expressed as a weighted sum over the island-restricted costs:

$$\mathcal{C}(p_{X_0}) = \sum_c p(c), \mathcal{C}(p_{X_0|c}), \tag{6}$$

It has been shown that each island-restricted cost function $\mathcal{C}(p_{X_0|c})$ admits a unique minimizer [1], denoted by

$$q^c_{X_0} = \arg \min_{r_0 \,:\, \mathrm{supp}(r_0) \subseteq \mathcal{Z}_c} \mathcal{C}(r_0).$$

A. Iterative method

In order to find the minimum of $\mathcal{C}(p_{X_0})$ in Eq. 2 subject to the constrain that $\sum_{x\in X} p_{X_0}(x) = 1$, we use the method of Lagrange multipliers. Evaluating the partial derivative of $\mathcal{C}(p_{X_0})$ with respect to $p_{X_0}(x')$ for an $x' \in X$;

$$\frac{\partial}{\partial p_{X_0}(x')}\mathcal{C}(p_{X_0}) = f(x') - \sum_{x\in X} G(x|x') \ln G p_{X_0}(x) + \ln p_{X_0}(x') \tag{7}$$

The normalization constraint $g(p_{X_0}) = \sum_x p_{X_0}(x) - 1 = 0$ introduces a Lagrange multiplier λ, and the prior distribution satisfies the following equation:

$$\frac{\partial}{\partial p_{X_0}(x')}\mathcal{C}(q_{X_0})\bigg|_{q_{X_0}} + \lambda \frac{\partial}{\partial p_{X_0}(x')} g(p_{X_0})\bigg|_{q_{X_0}} = 0 \tag{8}$$

This provides us an implicit equation for $q_{X_0}(x)$,

$$\ln q_{X_0}(x) + f(x) - \sum_{x'\in X} G(x'|x) \ln G q_{X_0}(x') + \lambda = 0, \tag{9}$$

which can also written as,

$$q_{X_0}(x) = e^{-\lambda - f(x)} \left[\prod_{x'\in\mathcal{X}} (G q_{X_0}(x'))^{G(x'|x)}\right]. \tag{10}$$

This equation is analytically solvable for only a few simple cases such as when G is a permutation or when G is complete erasure. However, for any G and $f(x)$, the solution of Eq. 10 can be found numerically. Consider the iterative version of Eq. 10,

$$q^{n+1}_{X_0}(x) = e^{-\lambda^n - f(x)} \left[\prod_{x'\in\mathcal{X}} (G q^{n}_{X_0}(x'))^{G(x'|x)}\right], \tag{11}$$

where λ^n ensuring that $q^{n+1}_{X_0}$ is normalized. The fixed point of Eq. 11 is unique when the map G has a single. It can be found numerically by starting with a random distribution and iteratively applying the map in 11 to generate a sequence of distribution. The sequence coverages towards to prior distribution.

B. Monte Carlo method

To minimize the cost function 2, we employ a simulated annealing algorithm that iteratively refines a candidate distribution over the simplex $\Delta_{\mathcal{X}}$ while gradually reducing randomness to ensure convergence. Start with a randomly initialized probability vector $q \in \Delta_{\mathcal{X}}$. Set the initial temperature T_0, cooling rate $\alpha < 1$, and interpolation parameter $s_0 \approx 1$. For each step $n = \{1, 2, \ldots\}$:

1. Sample a new random distribution $r \in \Delta_{\mathcal{X}}$.
2. Form a candidate distribution:
$$q' = (1 - s)r + sq,$$
and normalize if necessary.
3. Evaluate the cost:
$$\mathcal{C}(q') = S(Gq') - S(q') + \langle f\rangle_{q'}.$$
4. Accept q' with probability:
$$P = \begin{cases} 1, & \text{if } \mathcal{C}(q') < \mathcal{C}(q), \\ \exp\left(-\frac{\mathcal{C}(q') - \mathcal{C}(q)}{T}\right), & \text{otherwise.} \end{cases}$$
5. If accepted, set $q \leftarrow q'$ and update the best cost.

(a) Source Code (bubble sort)

```c
void bsort(int arr, int length) {
    bool swapped = true;
    int temp;
    int n = length;
    while (swapped) {
        swapped = false;
        for (int i = 0; i < n - 1; i++) {
            if (arr[i] > arr[i + 1]) {
                temp = arr[i];
                arr[i] = arr[i + 1];
                arr[i + 1] = temp;

                swapped = true;
            }
        }
        n = n - 1;
    }
}
```

(b) Lower-level Bubble Sort

```
00   LOAD R, array.         ; Input array
01   LOAD n, length         ; Total number of elements to sort → Rn
02   LOAD sw, 1             ; swapped = true

Start of while loop:

03   CMP sw, 0              ; while (swapped)
04   JE 18                  ; if swapped == false, exit sort

05   LOAD sw, 0             ; swapped = false
06   LOAD i, 1              ; i = 1

Start of inner loop:

07   CMP  i, n              ; Compare j with upper bound (shrinks with i)
08   JGE 17                 ; if i ≥ n, inner loop ends
09   CMP R[i], R[i+1]       ; Compare current and next elements
10   JLE 15                 ; If already in order, skip swap

Swap Block:

11   LOAD tmp, R[j]         ; Set R[j] = tmp2 (i.e., R[j+1])
12   LOAD R[j], R[j+1]      ; Set R[j+1] = tmp1 (i.e., R[j])
13   LOAD R[j+1], tmp       ; Set R[j+1] = tmp1 (i.e., R[j])
14   LOAD sw, 1             ; swapped = true

15   INC i                  ; i++
16   JMP 07                 ; Repeat inner loop

---------End of one outer loop pass--------

17   DEC n                  ; n = n - 1
18   JMP 03                 ; repeat if swapped == true

19   HALT                   ; Sorting complete
```

Supplementary Figure 1: (a) High-level source code for the bubble sort algorithm written in C. (b) Corresponding lower-level representation of the bubble sort program, showing how the values of all variables and the program counter evolve during execution. The input array is stored in register `R`, and its length in `n`. Registers `sw`, `i`, and `tmp` serve as scratch registers corresponding to the boolean flag `swapped`, the loop counter `i`, and the temporary variable `temp`, respectively. The full state of the program at any step is defined by the joint values of these registers along with the program counter. As instructions execute, both the register values and the program counter are updated, producing a sequence of state transitions.

6. Update the temperature: $T \leftarrow \alpha T$, and reduce s gradually.

The algorithm terminates after a fixed number of iterations or when successive updates no longer improve the cost. The final distribution q approximates the minimizer of $\mathcal{C}(p_{X_0})$. This annealing strategy ensures global exploration early on and local refinement as the temperature cools, making it suitable for non-convex optimization over the simplex. The implementation of both methods for optimizing the cost function, including simulated annealing and the fixed-point iteration, can be found at the following repository: `https://github.com/Kensho28/RASP/blob/main/RASPdag/prior_finding%20(1).ipynb`.

II. PROOFS

A. Lower bound on the worst-case mismatch cost

To derive a lower bound on the MMC as a function of the state-dependent term f, we focus on the case where the map G induces a single island decomposition. In this setting, the prior distribution q_{X_0} is unique.

The prior distribution q_{X_0} satisfies Eq. 9, derived using the method of Lagrange multipliers:

$$\ln q_{X_0}(x) + f(x) - \sum_{x' \in \mathcal{X}} G(x'|x) \ln \left[G q_{X_0}(x') \right] + \lambda = 0, \tag{12}$$

where λ is the Lagrange multiplier associated with the normalization constraint on q_{X_0}. Once we re-write $g(x) = \sum_{x' \in X} G(x'|x) \ln G q_{X_0}(x')$, the value of λ is given by,

$$\lambda = \ln \left(\sum_x \exp\left(g(x) - f(x) \right) \right). \tag{13}$$

Commands used in the RASP	
Symbol	Definition
`LOAD Rn, value`	Load a constant or memory value into register `Rn`
`STORE Rn, addr`	Store value in register `Rn` to memory address `addr`
`READ Rn, addr`	Read memory value at `addr` into register `Rn`
`ADD R1, R2, R3`	`R3` $\leftarrow$ `R1 + R2`
`SUB R1, R2, R3`	`R3` $\leftarrow$ `R1 - R2`
`MUL R1, R2, R3`	`R3` $\leftarrow$ `R1` $\times$ `R2`
`CMP R1, R2`	Compare `R1` and `R2`; set condition flags
`JMP addr`	Unconditional jump to address `addr`
`JGE addr`	Jump to `addr` if previous comparison was $\geq$
`JLE addr`	Jump to `addr` if previous comparison was $\leq$
`INC Rn`	Increment `Rn` by 1
`DEC Rn`	Decrement `Rn` by 1
`CALL addr`	Call subroutine at `addr`; push return address
`HALT`	Stop execution
`R[n]`	(Informal) Register array-like access notation

Supplementary Table I: Table of notation RASP commands used in the paper.

Note that $-\lambda$ corresponds to the residual cost. To see this, multiply both sides of Eq. 9 by $q_{X_0}(x)$ and sum over all $x \in \mathcal{X}$. This yields:

$$-\lambda = \sum_{x\in\mathcal{X}} q_{X_0}(x)\ln q_{X_0}(x) + \sum_{x\in\mathcal{X}} q_{X_0}(x) f(x) - \sum_{x'\in X} Gq_{X_0}(x')\ln Gq_{X_0}(x') \tag{14}$$

$$= -S(q_{X_0}) + \langle f\rangle_{q_{X_0}} + S(Gq_{X_0}) \tag{15}$$

$$= \mathcal{C}(q_{X_0}) \tag{16}$$

For any other initial distribution $p_{X_0} \in \Delta_{\mathcal{X}}$, the mismatch cost (MMC) is defined as the difference between the total cost incurred by p_{X_0} and the minimal cost achieved by the prior distribution q_{X_0}:

$$\mathrm{MC}(p_{X_0}) = \mathcal{C}(p_{X_0}) - \mathcal{C}(q_{X_0}). \tag{17}$$

Since the cost function $\mathcal{C}(p_{X_0})$ is convex over the probability simplex $\Delta_{\mathcal{X}}$, its maximum is attained at one of the vertices—that is, at a distribution $\delta_c \in \Delta_{\mathcal{X}}$ of the form:

$$\delta_c(x) = \begin{cases} 1 & \text{if } x = c, \\ 0 & \text{otherwise.} \end{cases} \tag{18}$$

At these corners, the cost evaluates to:

$$\mathcal{C}(\delta_c) = f(c) - \sum_{x\in\mathcal{X}} G(x|c)\ln G(x|c). \tag{19}$$

Since the term $-\sum_{x\in\mathcal{X}} G(x|c)\ln G(x|c)$ is upper bounded by $\ln|\mathcal{X}|$, it follows that if the function f satisfies the condition

$$\max_{x\in\mathcal{X}} f(x) - f(c) > \ln|\mathcal{X}|, \tag{20}$$

for any c such that $f(c) \neq \max_{x\in\mathcal{X}} f(x)$, then the maximum of the cost function over the corners is attained at the state where f achieves its maximum:

$$\arg\max_c \mathcal{C}(\delta_c) = \arg\max_c f(c). \tag{21}$$

This implies that the maximum of the cost function $\mathcal{C}$ is achieved at the vertex of the simplex corresponding to the state where $f(x)$ attains its maximum. Let us denote this state by

$$u = \arg\max_{x\in\mathcal{X}} f(x).$$

When condition 20 is satisfied, the maximum value of the cost function is given by:

$$\mathcal{C}(\delta_u) = f(u) - \sum_{x\in\mathcal{X}} G(x|u)\ln G(x|u), \tag{22}$$

Using Eqs. 13 and 22, we can now derive a lower bound on the worst-case mismatch cost:

$$\mathrm{MC}^* = \mathcal{C}(\delta_u) - \lambda, \tag{23}$$

$$= f(u) - \sum_{x\in\mathcal{X}} G(x|u)\ln G(x|u) + \ln\left(\sum_x \exp\left(g(x) - f(x)\right)\right), \tag{24}$$

where $g(x) := \sum_y G(y|x)\ln(Gq_{X_0}(y))$. Applying the log-sum-exp inequality,

$$\ln\left(\sum_x \exp(g(x) - f(x))\right) \geq \max_{x\in\mathcal{X}}\{g(x) - f(x)\}, \tag{25}$$

and noting that

$$\max_{x\in\mathcal{X}}\{g(x) - f(x)\} = -\min_{x\in\mathcal{X}}\{f(x) - g(x)\},$$

Since $-g(x) = -\sum_y G(y|x)\ln(Gq_{X_0}(y)) \leq \ln|\mathcal{X}|$, when the condition 20 is satisfied, the minimum can be written explicitly as

$$\min_{x\in\mathcal{X}}\{f(x) - g(x)\} = f(v) - g(v), \tag{26}$$

where $v = \arg\min_{x\in\mathcal{X}} f(x)$. Substituting this expression back into Eq. 23 yields:

$$\mathrm{MC}^* \geq f(u) - \sum_{x\in\mathcal{X}} G(x|u)\ln G(x|u) - f(v) + g(v). \tag{27}$$

We can also write this in terms of the max–min gap in f, giving:

$$\mathrm{MC}^* \geq \max_x f(x) - \min_x f(x) + g(v) - \sum_{x\in\mathcal{X}} G(x|u)\ln G(x|u). \tag{28}$$

Finally, we bound the last two terms using the entropy inequality:

$$-\ln|\mathcal{X}| \leq g_v - \sum_{x\in\mathcal{X}} G(x|u)\ln G(x|u) \leq \ln|\mathcal{X}|, \tag{29}$$

which leads to the worst-case lower bound:

$$\mathrm{MC}^* \geq \max_x f(x) - \min_x f(x) - \ln|\mathcal{X}|. \tag{30}$$

□

To illustrate this point, consider Fig. 2, which shows how the mismatch cost (MMC) contribution to total EP evolves as the function $f(x)$ is scaled by a factor k. We use the following stochastic map G:

$$G = \begin{bmatrix} 1-\phi & \phi & 0 \\ 0 & 1-\phi & \phi \\ \phi & 0 & 1-\phi \end{bmatrix}. \tag{31}$$

where $\phi \in [0, 1]$. Starting with small, arbitrary values of $f(x)$, increasing the scaling factor k causes the prior distribution induced by $kf(x)$ to shift toward a corner of the 2-simplex. This shift reflects increasingly peaked priors. Correspondingly, for a typical choice of initial distribution, the MMC becomes a progressively dominant component of the total EP, as demonstrated in Fig. 2.

Next, we examine how the MMC behaves under time coarse-graining. This analysis is crucial because MMC lower bounds can be established at various levels of temporal resolution. To take an example of Boolean circuits, MMC can be evaluated at the fine-grained level where each gate updates sequentially. Alternatively, a coarser time resolution may be adopted, in which groups of gates—such as layers in the circuit—are updated in parallel. At the coarsest level, the analysis considers only the initial and final configurations of the circuit.

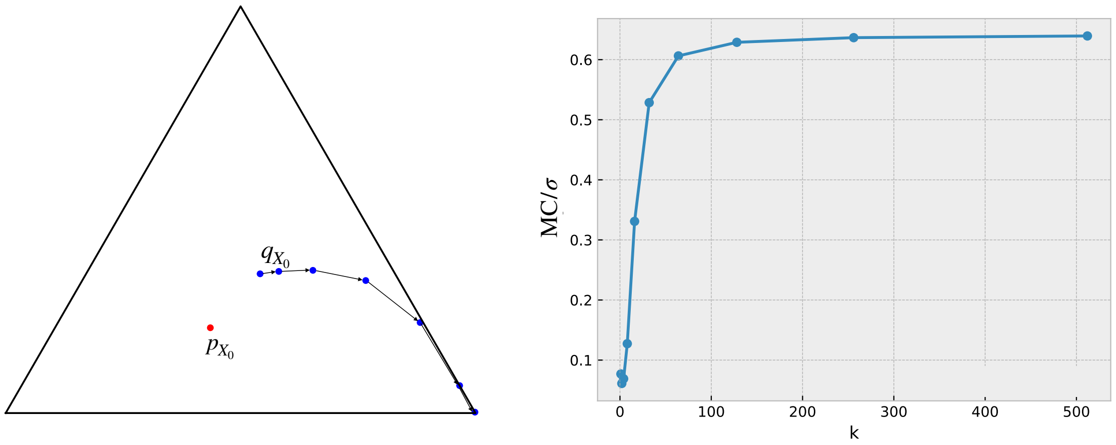

(a) (b)

Supplementary Figure 2: Large mismatch cost contribution in the high-EP regime: Starting with arbitrary chosen values $f(1) = 0.5$, $f(2) = 0.1$, and $f(3) = 0.2$, we scale all $f(x)$ by a factor k, resulting in new values $kf(x)$. (a) As k increases, the associated prior distribution $q_{X_0} = \arg\min_{r_{X_0}} \mathcal{C}(r_{X_0})$ (as defined by Eq. 2) shifts closer to the boundary of the 2-simplex. (b) Consequently, the mismatch cost for a typical initial distribution (here, $p_{X_0} = \{0.46, 0.33, 0.21\}$) becomes a larger fraction of the total EP, eventually exceeding 60% of the total EP. The calculations are performed using the map G defined in Eq. 31, with ϕ set to 0.1. The total entropy production is computed from $f(x)$ and $f(x)$ using Eq. 2, while the corresponding mismatch cost for each resulting prior is calculated using $\mathrm{MC}(p_{X_0}) = D(p_{X_0} \| q_{X_0}) - D(Gp_{X_0} \| Gq_{X_0})$.

B. Proof that prior is always in the interior of the simplex

Lemma A1 in the extended proofs below establishes that the optimal prior always lies in the interior of the simplex. The proof is adapted from [2], with minor corrections to fix typographical errors present in the original. For the reader's convenience, we reproduce the corrected version in full.

Consider a conditional distribution $P(y|x)$ that specifies the probability of "output" $y \in \mathcal{Y}$ given "input" $x \in \mathcal{X}$, where $\mathcal{X}$ and $\mathcal{Y}$ are finite.

Given some $\mathcal{Z} \subseteq \mathcal{X}$, the island decomposition $L_{\mathcal{Z}}(P)$ of P, and any $p \in \Delta_{\mathcal{X}}$, let $p(c) = \sum_{x \in c} p(x)$ indicate the total probability within island c, and

$$p^c(x) := \begin{cases} \frac{p(x)}{p(c)} & \text{if } x \in c \text{ and } p(c) > 0 \\ 0 & \text{otherwise} \end{cases} \tag{32}$$

indicate the conditional probability of state x within island c.

In our proofs below, we will make use of the notion of *relative interior*. Given a linear space V, the relative interior of a subset $A \subseteq V$ is defined as,

$$\operatorname{relint} A := \{x \in A : \forall y \in A, \exists \epsilon > 0 \text{ s.t. } x + \epsilon(x - y) \in A\}\,. \tag{33}$$

Finally, for any function $g(x)$, we use the notation

$$\partial_x^+ g(x)|_{x=a} := \lim_{\delta \to 0^+} \frac{1}{\delta}\left(g(a+\delta) - g(a)\right) \tag{34}$$

to indicate the right-handed derivative of $g(x)$ at $x = a$. When the condition that $x = a$ is omitted, a is implicitly assumed to equal 0, i.e.,

$$\partial_x^+ g(x) := \lim_{\delta \to 0^+} \frac{1}{\delta}\left(g(\delta) - g(0)\right) \tag{35}$$

We also adopt the shorthand that $a^\epsilon := a + \epsilon(b - a)$, and write $S(a^\epsilon) := S(p(a^\epsilon))$, $Pa^\epsilon := Pp(a^\epsilon)$, and so $S(Pa^\epsilon) = S(Pp(a^\epsilon))$.

Given some conditional distribution $P(y|x)$ and function $f : \mathcal{X} \to \mathbb{R}$, we consider the function $\mathcal{C} : \Delta_{\mathcal{X}} \to \mathbb{R}$ as

$$\mathcal{C}(p) := S(Pp) - S(p) + \mathbb{E}_p[f]\,. \tag{36}$$

Note that $\mathcal{C}$ is continuous on the relative interior of $\Delta_{\mathcal{X}}$.

Lemma A1. *For any $a, b \in \Delta_{\mathcal{X}}$, the directional derivative of $\mathcal{C}$ at a toward b is given by*

$$\partial^+_\epsilon \mathcal{C}(a + \epsilon(b-a))|_{\epsilon=0} = D(Pb\|Pa) - D(b\|a) + \mathcal{C}(b) - \mathcal{C}(a). \tag{37}$$

Proof. Using the definition of $\mathcal{C}$, write

$$\partial^+_\epsilon \mathcal{C}(a^\epsilon) = \partial^+_\epsilon \left[S(Pa^\epsilon) - S(a^\epsilon)\right] + \partial^+_\epsilon \mathbb{E}_{a^\epsilon}[f]. \tag{38}$$

Consider the first term on the RHS,

$$\begin{aligned}
&\partial^+_\epsilon \left[S(Pa^\epsilon) - S(a^\epsilon)\right] \\
&= -\sum_{y\in\mathcal{Y}} \left[(\partial^+_\epsilon Pa^\epsilon(y)) \ln Pa^\epsilon(y) + \partial^+_\epsilon [Pa^\epsilon](y)\right] \\
&\quad + \sum_{x\in\mathcal{X}} \left[(\partial^+_\epsilon a^\epsilon) \ln a^\epsilon(x) + \partial^+_\epsilon a^\epsilon(x)\right] \\
&= -\sum_{y\in\mathcal{Y}} (Pb(y) - Pa(y)) \ln Pa^\epsilon(y) + \sum_{x\in\mathcal{X}} (b(x) - a(x)) \ln a^\epsilon(x)
\end{aligned}$$

Evaluated at $\epsilon = 0$, the last line can be written as

$$\begin{aligned}
&-\sum_{y\in\mathcal{Y}} (Pb(y) - Pa(y)) \ln Pa(y) + \sum_{x\in\mathcal{X}} (b(x) - a(x)) \ln a(x) \\
&\quad = D(Pb\|Pa) + S(Pb) - S(Pa) - D(b\|a) - S(b) + S(a)
\end{aligned}$$

where we adopt the convention that if $a(x) = 0, b(x) \neq 0$ for some x, then this expression means $-\infty$. We next consider the $\partial^+_\epsilon \mathbb{E}_{a^\epsilon}[f]$ term,

$$\begin{aligned}
\partial^+_\epsilon \mathbb{E}_{a^\epsilon}[f] &= \partial^+_\epsilon \left[\sum_{x\in\mathcal{X}} (a(x) + \epsilon(b(x) - a(x)))\, f(x)\right] \\
&= \mathbb{E}_b[f] - \mathbb{E}_a[f]\,.
\end{aligned}$$

Combining the above gives

$$\begin{aligned}
\partial^+_\epsilon \mathcal{C}(a^\epsilon)|_{\epsilon=0} &= D(Pb\|Pa) - D(b\|a) + S(Pb) - S(b) \\
&\quad - (S(Pa) - S(a)) + \mathbb{E}_b[f] - \mathbb{E}_a[f] \\
&= D(Pb\|Pa) - D(b\|a) + \mathcal{C}(b) - \mathcal{C}(a).
\end{aligned}$$

□

Importantly, A1 holds even if there are x's for which $a(x) = 0$ but $b(x) \neq 0$, in which case the RHS of the equation in the lemma equals $-\infty$. (Similar comments apply to the results below.)

Theorem 1. *Let V be a convex subset of Δ. Then for any $q \in \arg\min_{s\in V} \mathcal{C}(s)$ and any $p \in V$,*

$$\mathcal{C}(p) - \mathcal{C}(q) \geq D(p\|q) - D(Pp\|Pq)\,. \tag{39}$$

Equality holds if q is in the relative interior of V.

Proof. Define the convex mixture $q^\epsilon := q + \epsilon(p - q)$. By A1, the directional derivative of $\mathcal{C}$ at q in the direction $p - q$ is

$$\partial^+_\epsilon \mathcal{C}(q^\epsilon)|_{\epsilon=0} = D(Pp\|Pq) - D(p\|q) + \mathcal{C}(p) - \mathcal{C}(q)\,.$$

At the same time, $\partial^+_\epsilon \mathcal{C}(q^\epsilon)|_{\epsilon=0} \geq 0$, since q is a minimizer within a convex set. 39 then follows by rearranging.

When q is in the relative interior of V, $q - \epsilon(p-q) \in V$ for sufficiently small $\epsilon > 0$. Then,

$$\begin{aligned} 0 &\le \lim_{\epsilon \to 0^+} \frac{1}{\epsilon}\left(\mathcal{C}(q - \epsilon(p-q)) - \mathcal{C}(q)\right) \\ &= - \lim_{\epsilon \to 0^-} \frac{1}{\epsilon}\left(\mathcal{C}(q + \epsilon(p-q)) - \mathcal{C}(q)\right) \\ &= - \lim_{\epsilon \to 0^+} \frac{1}{\epsilon}\left(\mathcal{C}(q + \epsilon(p-q)) - \mathcal{C}(q)\right) \\ &= -\partial_\epsilon^+ \mathcal{C}(q^\epsilon)|_{\epsilon=0}. \end{aligned}$$

where in the first inequality comes from the fact that q is a minimizer, in the second line we change variables as $\epsilon \mapsto -\epsilon$, and the last line we use the continuity of $\mathcal{C}$ on interior of the simplex. Combining with the above implies

$$\partial_\epsilon^+ \mathcal{C}(q^\epsilon) = D(Pp\|Pq) - D(p\|q) + \mathcal{C}(p) - \mathcal{C}(q) = 0.$$

□

The following result is key. It means that the prior within an island has full support in that island.

Lemma A2. *For any $c \in L(P)$ and $q \in \arg\min_{s:\text{supp } s \subseteq c} \mathcal{C}(s)$,*

$$\text{supp } q = \{x \in c : f(x) < \infty\}.$$

Proof. We prove the claim by contradiction. Assume that q is a minimizer with $\text{supp } q \subset \{x \in c : f(x) < \infty\}$. Note there cannot be any $x \in \text{supp } q$ and $y \in \mathcal{Y} \setminus \text{supp } Pq$ such that $P(y|x) > 0$ (if there were such an x, y, then $q(y) = \sum_{x'} P(y|x')q(x') \ge P(y|x)q(x) > 0$, contradicting the statement that $y \in \mathcal{Y} \setminus \text{supp } Pq$). Thus, by definition of islands, there must be an $\hat{x} \in c \setminus \text{supp } q$, $\hat{y} \in \text{supp } Pq$ such that $f(\hat{x}) < \infty$ and $P(\hat{y}|\hat{x}) > 0$.

Define the delta-function distribution $u(x) := \delta(x, \hat{x})$ and the convex mixture $q^\epsilon(x) = (1-\epsilon)q(x) + \epsilon u(x)$ for $\epsilon \in [0,1]$. We will also use the notation $q^\epsilon(y) = \sum_x P(y|x)q(x)$.

Since q is a minimizer of $\mathcal{C}$, $\partial_\epsilon \mathcal{C}(q^\epsilon)|_{\epsilon=0} \ge 0$. Since $\mathcal{C}$ is convex, the second derivative $\partial_\epsilon^2 \mathcal{C}(q^\epsilon) \ge 0$ and therefore $\partial_\epsilon \mathcal{C}(q^\epsilon) \ge 0$ for all $\epsilon \ge 0$. Taking $a = q^\epsilon$ and $b = u$ in A1 and rearranging, we then have

$$\begin{aligned} \mathcal{C}(u) &\ge D(u\|q^\epsilon) - D(Pu\|Pq^\epsilon) + \mathcal{C}(q^\epsilon) \\ &\ge D(u\|q^\epsilon) - D(Pu\|Pq^\epsilon) + \mathcal{C}(q), \end{aligned} \tag{40}$$

where the second inequality uses that q is a minimizer of $\mathcal{C}$. At the same time,

$$\begin{aligned} &D(u\|q^\epsilon) - D(Pu\|Pq^\epsilon) \\ &= \sum_y P(y|\hat{x}) \ln \frac{q^\epsilon(y)}{q^\epsilon(\hat{x})P(y|\hat{x})} \\ &= P(\hat{y}|\hat{x}) \ln \frac{q^\epsilon(\hat{y})}{\epsilon P(\hat{y}|\hat{x})} + \sum_{y \ne \hat{y}} P(y|\hat{x}) \ln \frac{q^\epsilon(y)}{\epsilon P(y|\hat{x})} \\ &\ge P(\hat{y}|\hat{x}) \ln \frac{(1-\epsilon)q(\hat{y})}{\epsilon P(\hat{y}|\hat{x})} + \sum_{y \ne \hat{y}} P(y|\hat{x}) \ln \frac{\epsilon P(y|\hat{x})}{\epsilon P(y|\hat{x})} \\ &= P(\hat{y}|\hat{x}) \ln \frac{(1-\epsilon)}{\epsilon} \frac{q(\hat{y})}{P(\hat{y}|\hat{x})}, \end{aligned} \tag{41}$$

where in the second line we've used that $q^\epsilon(\hat{x}) = \epsilon$, and in the third that $q^\epsilon(y) = (1-\epsilon)q(y) + \epsilon P(y|\hat{x})$, so $q^\epsilon(y) \ge (1-\epsilon)q(y)$ and $q^\epsilon(y) \ge \epsilon P(y|\hat{x})$.

Note that the RHS of 41 goes to ∞ as $\epsilon \to 0$. Combined with 40 and that $\mathcal{C}(q)$ is finite implies that $\mathcal{C}(u) = \infty$. However, $\mathcal{C}(u) = S(P(Y|\hat{x})) + f(\hat{x}) \le |\mathcal{Y}| + f(\hat{x})$, which is finite. We thus have a contradiction, so q cannot be the minimizer. □

The following result is also key. Intuitively, it follows from the fact that the directional derivative of $S(p)$ into the simplex for any p on the edge of the simplex is negative infinite.

Lemma A3. *For any island $c \in L(P)$, $q \in \arg\min_{s:\text{supp } s \subseteq c} \mathcal{C}(p)$ is unique.*

Proof. Consider any two distributions $p, q \in \arg\min_{s:\mathrm{supp}\, s \subseteq c} \mathcal{C}(s)$, and let $p' = Pp$, $q' = Pq$. We will prove that $p = q$.

First, note that by A2, supp q = supp $p = c$. By 1,

$$\begin{aligned}\mathcal{C}(p) - \mathcal{C}(q) &= D(p\|q) - D(p'\|q') \\ &= \sum_{x,y} p(x)P(y|x) \ln \frac{p(x)q'(y)}{q(x)p'(y)} \\ &= \sum_{x,y} p(x)P(y|x) \ln \frac{p(x)P(y|x)}{q(x)p'(y)P(y|x)/q'(y)} \\ &\geq 0\end{aligned}$$

where the last line uses the log-sum inequality. If the inequality is strict, then p and q can't both be minimizers, i.e., the minimizer must be unique, as claimed.

If instead the inequality is not strict, i.e., $\mathcal{C}(p) - \mathcal{C}(q) = 0$, then there is some constant α such that for all x, y with $P(y|x) > 0$,

$$\frac{p(x)P(y|x)}{q(x)p'(y)P(y|x)/q'(y)} = \alpha \tag{42}$$

which is the same as

$$\frac{p(x)}{q(x)} = \alpha \frac{p'(y)}{q'(y)}. \tag{43}$$

Now consider any two different states $x, x' \in c$ such that $P(y|x) > 0$ and $P(y|x') > 0$ for some y (such states must exist by the definition of islands). For 43 to hold for both x, x' with that same, shared y, it must be that $p(x)/q(x) = p(x')/q(x')$. Take another state $x'' \in c$ such that $P(y'|x'') > 0$ and $P(y'|x') > 0$ for some y'. Since this must be true for all pairs $x, x' \in c$, $p(x)/q(x) =$ const for all $x \in c$, and $p = q$, as claimed. □

Lemma A4. $\mathcal{C}(p) = \sum_{c \in L(P)} p(c)\mathcal{C}(p^c)$.

Proof. First, for any island $c \in L(P)$, define

$$\phi(c) = \{y \in \mathcal{Y} : \exists x \in c \text{ s.t. } P(y|x) > 0\}.$$

In words, $\phi(c)$ is the subset of output states in $\mathcal{Y}$ that receive probability from input states in c. By the definition of the island decomposition, for any $y \in \phi(c)$, $P(y|x) > 0$ only if $y \in c$. Thus, for any p and any $y \in \phi(c)$, we can write

$$\frac{Pp(y)}{p(c)} = \frac{\sum_x P(y|x)p(x)}{p(c)} = \sum_{x \in \mathcal{X}} P(y|x)p^c(x). \tag{44}$$

Using $p(x) = \sum_{c \in L(P)} p(c)p^c(x)$ and linearity of expectation, write $\mathbb{E}_p[f] = \sum_{c \in L(P)} p(c)\mathbb{E}_{p^c}[f]$. Then,

$$\begin{aligned}&S(Pp) - S(p) \\ &= -\sum_y Pp(y) \ln Pp(y) + \sum_x p(x) \ln p(x) \\ &= \sum_{c \in L(P)} p(c) \Big[-\sum_{y \in \phi(c)} \frac{Pp(y)}{p(c)} \ln \frac{Pp(y)}{p(c)} + \sum_{x \in c} \frac{p(x)}{p(c)} \ln \frac{p(x)}{p(c)} \Big] \\ &= \sum_{c \in L(P)} p(c) \left[S(Pp^c) - S(p^c) \right],\end{aligned}$$

where in the last line we've used 44. Combining gives

$$\begin{aligned}\mathcal{C}(p) &= \sum_{c \in L(P)} p(c) \left[S(Pp^c) - S(p^c) + \mathbb{E}_{p^c}[f] \right] \\ &= \sum_{c \in L(P)} p(c)\mathcal{C}(p^c).\end{aligned}$$

□

We are now ready to prove the main result of this appendix.

Theorem 2. *Consider any function $\mathcal{C} : \Delta_{\mathcal{X}} \to \mathbb{R}$ of the form*

$$\mathcal{C}(p) := S(Pp) - S(p) + \mathbb{E}_p[f]$$

where $P(y|x)$ is some conditional distribution of $y \in \mathcal{Y}$ given $x \in \mathcal{X}$ and $f : \mathcal{X} \to \mathbb{R} \cup \{\infty\}$ is some function. Let $\mathcal{Z}$ be any subset of $\mathcal{X}$ such that $f(x) < \infty$ for $x \in \mathcal{Z}$, and let $q \in \Delta_{\mathcal{Z}}$ be any distribution that obeys

$$q^c \in \arg \min_{r:\mathrm{supp}\ r \subseteq c} \mathcal{C}(r) \quad \textit{for all} \quad c \in L_{\mathcal{Z}}(P).$$

Then, each q^c will be unique, and for any p with $\mathrm{supp}\ p \subseteq \mathcal{Z}$*,*

$$\mathcal{C}(p) = D(p\|q) - D(Pp\|Pq) + \sum_{c \in L_{\mathcal{Z}}(P)} p(c)\mathcal{C}(q^c).$$

Proof. We prove the theorem by considering two cases separately.

Case 1: $\mathcal{Z} = \mathcal{X}$. This case can be assumed when $f(x) < \infty$ for all x, so that $L_{\mathcal{Z}}(P) = L(P)$. Then, by A4, we have $\mathcal{C}(p) = \sum_{c \in L(P)} p(c)\mathcal{C}(p^c)$. By A2 and 1,

$$\mathcal{C}(p^c) - \mathcal{C}(q^c) = D(p^c\|q^c) - D(Pp^c\|Pq^c),$$

where we've used that if some $\mathrm{supp}\ q^c = c$, then q^c is in the relative interior of the set $\{s \in \Delta_{\mathcal{X}} : \mathrm{supp}\ s \subseteq c\}$. q^c is unique by A3.

At the same time, observe that for any $p, r \in \Delta_{\mathcal{X}}$,

$$\begin{aligned}
&D(p\|r) - D(Pp\|Pr)\\
&= \sum_x p(x) \ln \frac{p(x)}{r(x)} - \sum_y Pp(y) \ln \frac{Pp(y)}{Pr(y)}\\
&= \sum_{c \in L(P)} p(c) \Bigg[\sum_{x \in c} \frac{p(x)}{p(c)} \ln \frac{p(x)/p(c)}{r(x)/r(c)}\\
&\qquad\qquad - \sum_{y \in \phi(c)} \frac{Pp(y)}{p(c)} \ln \frac{Pp(y)/p(c)}{Pr(y)/r(c)} \Bigg]\\
&= \sum_{c \in L(P)} p(c) \left[D(p^c\|r^c) - D(Pp^c\|Pr^c) \right] .
\end{aligned}$$

The theorem follows by combining.

Case 2: $\mathcal{Z} \subset \mathcal{X}$. In this case, define a "restriction" of f and P to domain $\mathcal{Z}$ as follows:

1. Define $\tilde{f} : \mathcal{Z} \to \mathbb{R}$ via $\tilde{f}(x) = f(x)$ for $x \in \mathcal{Z}$.
2. Define the conditional distribution $\tilde{P}(y|x)$ for $y \in \mathcal{Y}, x \in \mathcal{Z}$ via $\tilde{P}(y|x) = P(y|x)$ for all $y \in \mathcal{Y}, x \in \mathcal{Z}$.

In addition, for any distribution $p \in \Delta_{\mathcal{X}}$ with $\mathrm{supp}\ p \subseteq \mathcal{Z}$, let $\tilde{p}$ be a distribution over $\mathcal{Z}$ defined via $\tilde{p}(x) = p(x)$ for $x \in \mathcal{Z}$. Now, by inspection, it can be verified that for any $p \in \Delta_{\mathcal{X}}$ with $\mathrm{supp}\ p \subseteq \mathcal{Z}$,

$$\mathcal{C}(p) = S(\tilde{P}\tilde{p}) - S(\tilde{p}) + \mathbb{E}_{\tilde{p}}[\tilde{f}] =: \tilde{\mathcal{C}}(\tilde{p}) \tag{45}$$

We can now apply Case 1 of the theorem to the function $\tilde{\mathcal{C}} : \Delta_{\mathcal{Z}} \to \mathbb{R}$, as defined in terms of the tuple $(\mathcal{Z}, \tilde{f}, \tilde{P})$ (rather than the function $\mathcal{C} : \Delta_{\mathcal{X}} \to \mathbb{R}$, as defined in terms of the tuple $(\mathcal{X}, f, P)$). This gives

$$\tilde{\mathcal{C}}(\tilde{p}) = D(\tilde{p}\|\tilde{q}) - D(\tilde{P}\tilde{p}\|\tilde{P}\tilde{q}) + \sum_{c \in L(\tilde{P})} \tilde{p}(c)\tilde{\mathcal{C}}(\tilde{q}^c), \tag{46}$$

where, for all $c \in L(\tilde{P})$, $\tilde{q}^c$ is the unique distribution that satisfies $\tilde{q}^c \in \arg\min_{r \in \Delta_{\mathcal{Z}}:\mathrm{supp}\ r \subseteq c} \tilde{\mathcal{C}}(r)$.

Now, let q be the natural extension of $\tilde{q}$ from $\Delta_{\mathcal{Z}}$ to $\Delta_{\mathcal{X}}$. Clearly, for all $c \in L(\tilde{P})$, $\mathcal{C}(q^c) = \tilde{\mathcal{C}}(\tilde{q}^c)$ by 45. In addition, each q^c is the unique distribution that satisfies $q^c \in \arg\min_{r \in \Delta_{\mathcal{X}}:\mathrm{supp}\, r \subseteq c} \mathcal{C}(r)$. Finally, it is easy to verify that $D(\tilde{p}\|\tilde{q}) = D(p\|q)$, $D(\tilde{P}\tilde{p}\|\tilde{P}\tilde{q}) = D(Pp\|Pq)$, $L(\tilde{P}) = L_{\mathcal{Z}}(P)$. Combining the above results with 45 gives

$$\mathcal{C}(p) = \tilde{\mathcal{C}}(\tilde{p}) = D(p\|q) - D(Pp\|Pq) + \sum_{c \in L_{\mathcal{Z}}(P)} p(c)\mathcal{C}(q^c).$$

□

[1] D. H. Wolpert and A. Kolchinsky, New Journal of Physics **22**, 063047 (2020).
[2] A. Kolchinsky and D. H. Wolpert, Physical Review Research **2**, 033312 (2020).
